# A systematic review of current national hospital-based stroke registries monitoring access to evidence-based care and patient outcomes

**DOI:** 10.1177/23969873241311821

**Published:** 2025-01-21

**Authors:** Chloe Leigh, Jodie Gill, Zainab Razak, Shirsho Shreyan, Dominique A Cadilhac, Joosup Kim, Natasha A Lannin, Martin Dennis, Moira Kapral, Jeyaraj Pandian, Yudi Hardianto, Beilei Lin, Atte Meretoja, Noor Azah Abd Aziz, Lee Schwamm, Bo Norrving, Lekhjung Thapa, Marshall Dozier, Shyam Kelavkar, Gillian Mead

**Affiliations:** 1 University of Edinburgh, Edinburgh, UK; 2 Rajshahi Medical College, Laxmipur Rajpara, Bangladesh; 3 Department of Medicine, School of Clinical Sciences at Monash Health, Monash University, Victorian Heart Hospital, Clayton, VIC, Australia; 4 Brain Recovery and Rehabilitation Group, Department of Neuroscience, School of Translational Medicine, Monash University, Melbourne, VIC, Australia; 5 Centre for Clinical Brain Sciences, University of Edinburgh, Edinburgh, UK; 6 Department of Medicine, University of Toronto, Toronto, ON, Canada; 7 Christian Medical College, Ludhiana, Punjab, India; 8 Faculty of Nursing, Hasanuddin University, Makassar, Indonesia; 9 School of Nursing and Health, Zhengzhou University, Zhengzhou, Henan, China; 10 Helsinki University Hospital, Helsinki, Finland; 11 Department of Family Medicine, Medical Faculty, National University of Malaysia, UKM Medical Centre Cheras, Kuala Lumpur, Malaysia; 12 Yale School of Medicine, New Haven, CT, USA; 13 Department of Clinical Sciences, Skåne University Hospital, Lund University, Lund, Sweden; 14 National Neuro Centre, Kathmandu, Nepal; 15 Information Services, Medicine and Veterinary Medicine, Library and University Collections, University of Edinburgh, Edinburgh, UK; 16 Addenbrookes Hospital, Cambridge, UK; 17 Stroke and Elderly Care Medicine, University of Edinburgh, Edinburgh, UK

**Keywords:** Stroke, registry, audit, quality

## Abstract

**Background:**

National stroke clinical quality registries/audits support improvements in stroke care. In a 2016 systematic review, 28 registries were identified. Since 2016 there have been important advances in stroke care, including the development of thrombectomy services. Therefore, we sought to understand whether registries have evolved with these advances in care. The aim of this systematic review was to identify current, hospital-based national stroke registries/audits and describe variables (processes, outcome), methods, funding and governance).

**Methods:**

We searched four databases (21st May 2015 to 1st February 2024), grey literature and stroke organisations’ websites. Initially two reviewers screened each citation; when agreement was satisfactory, one of four reviewers screened each citation. The same process was applied to full texts. If there were no new publications from registries identified in the original 2016 review, we contacted the registry leads. We extracted data using predefined categories on country (including income level), clinical/process variables, methods, funding and governance.

**Results:**

We found 37 registries from 31 countries (28 high income, four upper-middle income, five lower-middle income) of which 16 had been identified in 2016 and 21 were new. Twenty-two of the same variables were collected by >50% of registries/audits (mostly acute care, including thrombectomy, and secondary prevention), compared with only four variables in 2016. Descriptions of funding, management, methods of consent and data privacy, follow-up, feedback to hospitals, linkage to other datasets and alignment of variables with guidelines were variably reported. Reasons for apparent termination of some registries was unclear.

**Conclusions:**

The total number of stroke registries has increased since 2016, and the number of variables collected has increased, reflecting advances in stroke care. However, some registries appeared to have ceased; the reasons are unclear.

## Graphical abstract

**Graphical abstract:**
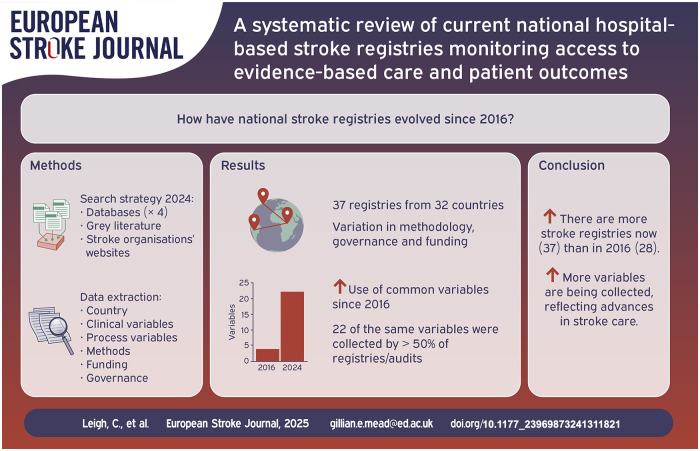

## Introduction

National stroke registries or audits are comprehensive databases that monitor a country’s hospital performance in providing stroke treatments.^1^ Registries provide insights into stroke care systems, facilitate analysis of treatment quality between centres, identify inequalities in stroke care, and facilitate quality improvement and research.

The first systematic review describing the characteristics of, and variables collected in, 28 national stroke registries from 26 countries was published in 2016 by Cadilhac et al.^1^ Since then, there have been important advances in stroke care, including thrombectomy, and registries should have evolved to reflect these changes. Thayabaranathan et al.^2^ reported global stroke statistics in 2022; and identified seven new registries (Israel, Switzerland, Spain, Netherlands, Norway, Czech Republic and India), but did not report whether all the 2016 registries were still ongoing and whether they had evolved.

To determine whether current stroke registries reflect advances in stroke care since 2016, we aimed to update the review by Cadilhac et al.^1^ by systematically identifying current stroke registries, describing their characteristics including data variables collected, methodology, on funding and governance.

## Methods

We registered our protocol for this updated review with INPLASY on 22 October 2023 (Supplementary Material). Our literature search adhered to the PRISMA 2020 Statement.^3^ We built on the previous search strategy^1^ (Supplementary Material) to perform a comprehensive electronic search of literature published between 21 May 2015 and 1 February 2024 (Ovid Medline, Embase and Global Health; and WHO Global Index Medicus).

Search terms were: (((Ischemic Attack, Transient) OR (Stroke) OR (Cerebral Hemorrhage) OR ((Ischemic adj2 (stroke OR attack) or acute stroke) AND Registries)) OR ((national OR central*) adj5 stroke adj5 regist*) OR (stroke and audit)) OR (stroke AND (Internet OR web) adj2 data collection).

We identified grey literature through websites of World Stroke Organisation (WSO), European Stroke Organisation (ESO), African Stroke Organisation (ASO) and American Heart Association (AHA). We checked existing websites described in the 2016 review.

We contacted authors of registries from the 2016 review that appeared to be no longer ongoing (Supplemental Table 3).

### Inclusion criteria

We used the AHA’s definition of stroke, excluding subarachnoid haemorrhage.^4^ A national stroke registry or audit is a database used to report clinical stroke-care indicators for consecutive patients hospitalised for acute stroke, within a defined population.^1^ ‘National’ means country-wide data collection, even if full coverage had not yet been achieved, providing this was the stated intent. We classified a country as a member state of the United Nations (UN) or a constituent country of a UN member state.^5^ Devolved nations were included. ‘Current’ was defined as a new publication since 1 January 2016 or registry lead confirmed it was still ongoing. Registries had to have collected at least one full year of acute stroke data.

### Exclusion criteria

We excluded registries reporting a small subgroup of patients, for example, mechanical thrombectomy. Those that collected data for only epidemiological understanding or incidence reporting, registries that were not stroke specific and those that did not collect data about acute stroke care.

### Selection of registry programs

We completed deduplication automatically through Covidence then manually. For the first 619 of 7568 (8.2%) titles/abstracts, a junior reviewer (CL, JG or ZR) and a senior reviewer (GEM) screened each one. Because no relevant citations were missed by the junior reviewer, one reviewer (CL, JG, ZR or GEM) screened the remaining 6946 citations. We obtained full texts for potentially eligible studies; two reviewers (including GEM) scrutinized the first 429 of 1138 papers (38%), then a single reviewer the rest (because the junior reviewer had not missed any relevant papers).

### Data extraction

We used the same methods as Cadhilac et al.^1^ We used Google Translate for non-English publications. JG, ZR, or CL extracted data about governance, funding, hospital participation and coverage (Supplemental Table 4), and variables (Table 2 and Supplemental Table 5) respectively from all available sources. For each registry we cited one paper. Table 1 data were checked by SS. We divided the registries into income level (World Bank Classification).^6^

**Table 1.** Stroke registry methods.

Registry	Active dates	Web-based data collection?	Consent-opt out/waiver	Follow-up?	Targets/guidelines used to benchmark performance?	Feedback to hospitals?	Methods to achieve data completeness?	Anonymised data?	Data linkage
Australia: Stroke Clinical Registry^7^	2009–	✔	✔	✔	✔	✔	✔	✔	✔
Australia: National Audit of Acute Services^8^	2007–	✔	Consent forms for hospitals	NS	✔	✔	✔	✔	✔
Austria^9^	2003–	✔	✔	✔	NS	NS	✔	✔	NS
Barbados^10^	2008–	✔	NS	NS	✔	NS	NS	✔	✔
China: Stroke Centre Alliance Programme^11^	2015–2019	✔	✔	No follow-up	✔	✔	✔	✔	No – has the potential
China: Bigdata Observatory Platform^12^	2011–	NS	NS	NS	NS	NS	NS	NS	NS
China: third National Stroke Registry^13^	2015–2018	✔	✔	✔	NS	✔	✔	Password protected electronic system	NS
Czech Republic (RES-Q)^14^	2016–	✔	NS	NS	✔	✔	NS	NS	✔
Denmark^15^	2003–	✔	✔	✔	NS	NS	✔	NS	✔
Finland (PERFECT Stroke)^16^	1999–	✔	✔	✔	No target setting	✔	✔	✔	✔
Germany^17^	1999–	✔	✔ but informed consent for follow-up	NS	NS	NS	✔	NS	NS
Hungary (RES-Q)^18^	2016–	✔	NS	NS	✔	✔	NS	NS	✔
India^19^	2018–2019	Forms	NS	✔	NS	NS	✔	NS	NS
Ireland^20^	2011–	✔	NS	No follow-up	✔	✔	✔	✔	NS
Israel: National Acute Stroke Israeli Survey Registry^21^	2004–2016	✔	✔	NS	NS	NS	NS	✔	NS
Israeli National Stroke Registry^22^	2014–	✔	✔	NS	NS	NS	NS	✔	✔
Japan^23^	1999–	✔	✔	NS	✔	No	NS	✔	No – aims to in the future
Kyrgyzstan (RES-Q)^24^	2019–	✔	NS	NS	✔	✔	NS	NS	✔
Malaysia^25^	2009–	✔	NS	✔	✔	NS	NS	✔	NS
Nepal (RES-Q)^26^	2021–	✔	NS	NS	✔	✔	NS	NS	✔
The Netherlands^27^	2014–	✔	✔	✔	✔	✔	NS	✔	✔
Norway^28^	2007–	✔	✔	✔	✔	NS	✔	✔	NS
Qatar^29^	2014–	NS	✔	✔	✔	NS	✔	NS	NS
Romania (RES-Q)^30^	2017–	✔	NS	NS	✔	✔	NS	NS	✔
Scotland^31^	2002–	✔	✔	NS	✔	✔	✔	✔	✔
Singapore^32^	2002–	✔	✔	NS	NS	NS	NS	NS	NS
Slovakia^33^	2010–	NS	✔	NS	✔	NS	NS	✔	NS
South Korea^34^	2006–	✔	✔	✔	NS	✔	NS	✔	NS
Spain^35^	2011–2019	NS	NS	NS	NS	NS	NS	NS	NS
Sri Lanka^36^	2016–2017	✔	✔	✔	✔	✔	✔	Password protected electronic system	NS
Sweden^37^	1994–	✔	✔	✔	✔	✔	✔	✔	✔
Switzerland^38^	2014–	✔	NS	✔	✔	NS	NS	✔	✔
Taiwan^39^	2006–	✔	Signed informed consent forms	✔	NS	NS	✔	✔	NS
UK – England, Wales and Northern Ireland^40^	2013–	✔✔	NS	✔	✔	✔	NS	✔	✔
USA Get With The Guidelines^41^	2003–	✔	✔	✔	✔	✔	✔	NS	No
USA. Paul Coverdell National Acute Stroke Program^42^	2001–	✔	NS	✔	✔	✔	✔	NS	✔
Uzbekistan^43^	2019–2021	NS	NS	NS	NS	NS	NS	NS	NS

NS: not specified.

There are no relevant risk of bias tools, so we narratively reviewed methodological quality.

## Results

### Search results

**Figure 1.**
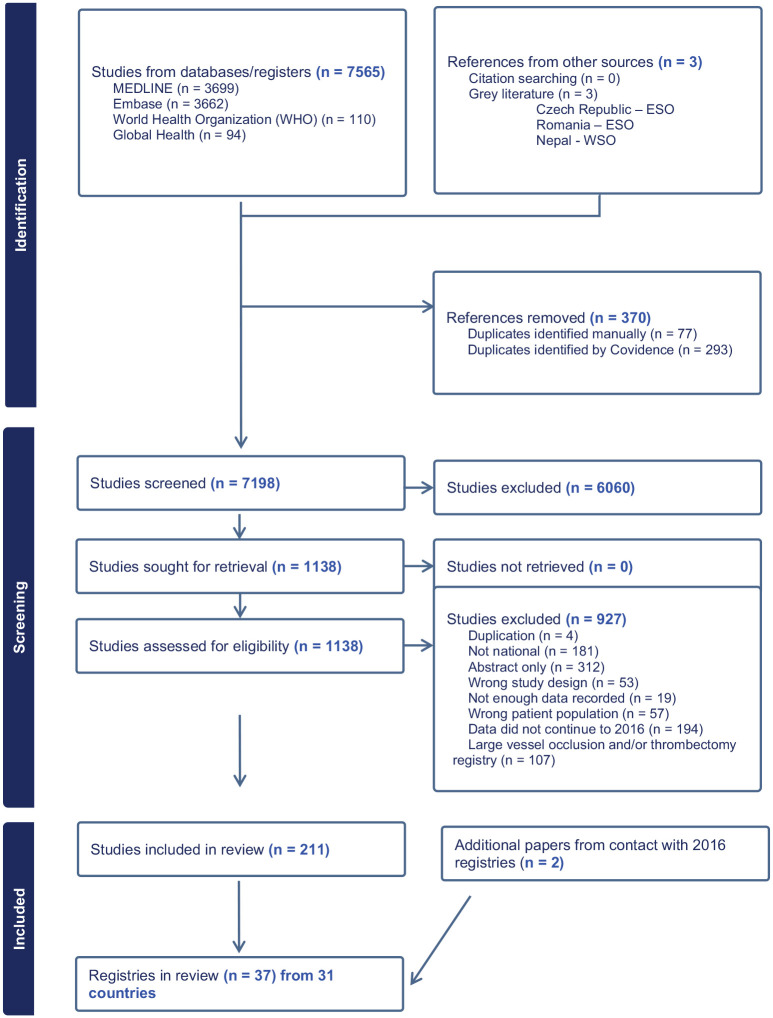
Prisma flow-diagram showing results of the searches.

Electronic searches identified 7565 titles/abstracts (including 370 duplicates). A further three registries (Romania, Czech Republic, Nepal) were identified in grey literature. Of the 1138 full texts retrieved, we included 211 papers (Figure 1) reporting 36 national stroke registries from 31 countries (Table 1 and Supplemental Table 5). One further registry (Finland) identified in 2016 was confirmed as ongoing by the registry lead, making a total of 37 registries from 32 countries.^7–43^ Reasons for exclusion are provided in Table 2 (Supplementary Materials). Of the 28 registries identified in 2016, 16 were identified again in 2024, and 11 appeared to be no longer active. South Korea had been reported in 2016 as having two separate registries; in 2024, there was just one. Of the 11 no longer active, one author (Argentina) responded and indicated that it had been a time-limited registry. We identified 21 new registries.

**Table 2.** List of variables collected by >50% of registries in 2024.

Age	Door to needle time
Sex	Time to neuroimaging
Stroke severity	Access to thrombectomy
Primary functional status	**Antithrombotic therapy during hospitalisation**
Previous stroke	Length of stay
Comorbidity	Rehabilitation
Smoking	**Stroke unit care**
Brain or vascular imaging	Dysphagia screening
Known time of symptom onset	**Discharged on antithrombotic medication**
Mode of admission	Discharged on statin/lipid-lowering medication
Onset to door time	Functional Status follow-up
**Thrombolysis**	Survivor status

The variables in bold were collected by >50% of registries in 2016.

### Included registries

National stroke registries were identified in Asia, Europe, North America and Oceania (Table 1). Some countries use the term audit and registry interchangeably. For registries collecting data only for a defined period each year (e.g. a 2 month period for the Australian Audit^8^), we included them providing there was a year’s worth of data in total. China maintains three different national registries;^11–13^ Israel, USA and Australia each have two. The rest each have one. Twenty-eight were in high-income countries (HICs); four (11%) in upper-middle-income countries (UMICs) (three in China, one in Malaysia); and five (14%) in lower-middle-income countries (LMICs) (Nepal, Uzbekistan, India, Sri Lanka and Kyrgyzstan) and none from low-income countries (LICs).

Methods are summarized in Table 1. More than half of the registries used web-based data collection, waiver of consent, performed at least one follow-up, measured performance against guidelines/targets (usually from their own countries), provided feedback from hospitals, and performed data checks. Data linkage was performed by 16 registries.

### Funding (Supplementary Materials)

Seventeen (46%) registries were funded by their government/government health service, 11 had several funding sources, and the others were Registry of Stroke Care Quality (RES-Q) or unspecified funding sources. Several reported the exact amount of funding received.

### Management and governance (Supplemental Table 4)

‘Management’ meant organization and direction of workflow and operations; this judgement often required the scrutinization of multiple publications for each registry. In both HICs and MICS, 17 were managed by the Government/Government Health Service, and the funding source mostly matched the managing organization. Eleven registries without funding were guided by another organization.

A steering committee/group, comprising expert individuals was the most common type of governance (15) registries from both MICs and HICs. Other common types of governance included a scientific committee (5), an advisory group/committee (3), and a management committee (2). Registries in HICs had either voluntary (12) mandatory (8) or unspecified participation (8). Registry coverage was the least reported aspect of organization (eight registries in HICs).

The number of hospitals participating in their national registries was related to the size of the country. For example, Qatar’s national stroke registry only includes one major hospital covering 90% of acute strokes.^29^ In India, two registries collect data from regional registries or major regional hospitals and consolidate them into a single registry. The Austrian stroke registry only included hospitals with stroke units.^9^

### Variables collected (Supplemental Tables 6–8)

Full information about data variables was unavailable for 12 (32%) registries. Those maintained by RES-Q collected the same variables. Of 58 variables collected, the number ranged from six^33^ to 37.^34^ Of eight patient variables collected, the number ranged from one^33^ to eight.^12,21,41^ Of 37 process data variables recorded, these ranged from two^43^ to 23.^25,37,41^ Mechanical thrombectomy access was recorded in 25 (69%) registries. Three (8%) registries reported aggressive blood pressure lowering for acute intracerebral haemorrhage.^25,40,41^ The number of outcome metrics reported ranged from none^33^ to eight.^7^

In 2016, only four variable categories were collected by >50% registries (intravenous thrombolysis, antithrombotic therapy during hospitalization, discharge on antithrombotic medication and stroke unit management); in 2024, an additional 18 variables were collected by >50% of registries (Table 2).

## Discussion

This updated systematic review identified 37 national registries from 32 countries, by screening 7565 citations and scrutinizing 1138 full texts. Sixteen registries had been identified in 2016, and 21 registries were new. The number of clinical/process variables collected (Table 2) has increased since 2016. One registry active in 2016 is definitely no longer active. Ten registries identified in 2016 appeared to be no longer active (i.e. no recent publications, and no response from the authors when we sought clarification). Possible reasons are that they have been replaced by registries with new names, or have started to use multinational platforms for data collection including RES-Q, a worldwide stroke care quality improvement platform that collects, analyses, visualizes, benchmarks stroke care practices and outcomes around the world.

All 37 registries aimed to improve stroke care and thus outcomes from stroke by reporting process of care, but there were gaps in the reporting several important aspects of care (‘not stated’ in Table 1). We found gaps in reporting of funding, governance, how consent was obtained and patient follow-up. Those registries requiring consent risk under-inclusion of people with lower health literacy.^44^ Rehabilitation and long-term planning variables were less frequently collected than those for hyperacute care and secondary prevention, though we excluded registries focusing only on rehabilitation. The registries we identified did not report recent innovations include mobile stroke units.

We used the same methods has had been used in the 2016 systematic review.^3^ We improved the sensitivity of previous searches. However, some screening was done by only one reviewer, and data were extracted by one (though a second author checked Table 1 data). We only included registries with current or intended national coverage. We did not report important regional registries. Another limitation was identification of registries through peer-reviewed scientific publications only. Generally, when a quality improvement registry is established, it takes several years to collect and publish data internationally. Most improvement work occurs nationally, in a national language, outside peer review processes. We extracted data from scientific publications and the national reports that we identified through our searches. Relevant information might have been available about the registries from sources that we could not access. Direct follow-up with the registries themselves may have been a more effective way of doing this; we were able to do this for registries led by authors of this systematic review, but we did not have the resources to do this for all registries. This might have introduced bias in the amount of information available for each registry.

We acknowledge that some national registries may have been missed, including countries using RES-Q and we assumed that if a registry was identified in our searches (from May 2015) that it was still current.

There are implications for clinical services. Countries without registries could use this review to help design their own registries, or use the RES-Q platform.^45^ RES-Q is now used in over 2000 hospitals in 92 countries.^17^ In many LMICs, this may provide their only possibility of a national registry. For future narrative reviews, this RES-Q register could be scrutinized. This was not pre-specified in our protocol but should be considered for future updates of this review.

Other countries which may have national registries, but no peer reviewed publications, should consider publishing their work, so that data are easily available in the public domain; although arguably government level data need not be shared in this way. There could be more consistent, complete, transparent reporting of registry design. National stroke organisations could develop standardized lists of core variables (including process and outcome variables) mapped to stroke guidelines, to enable international comparisons and better understanding quality of stroke-care systems, though registries should have flexibility to collect data specific to the needs of the setting. An international repository, with up-to-date information about stroke registries would allow clinicians and researchers interested in registries to collaborate more closely, even if registry authors do not publish data in scientific journals.

## Supplemental Material

sj-docx-1-eso-10.1177_23969873241311821 – Supplemental material for A systematic review of current national hospital-based stroke registries monitoring access to evidence-based care and patient outcomes Supplemental material, sj-docx-1-eso-10.1177_23969873241311821 for A systematic review of current national hospital-based stroke registries monitoring access to evidence-based care and patient outcomes by Chloe Leigh, Jodie Gill, Zainab Razak, Shirsho Shreyan, Dominique A Cadilhac, Joosup Kim, Natasha A Lannin, Martin Dennis, Moira Kapral, Jeyaraj Pandian, Yudi Hardianto, Beilei Lin, Atte Meretoja, Noor Azah Abd Aziz, Lee Schwamm, Bo Norrving, Lekhjung Thapa, Marshall Dozier, Shyam Kelavkar and Gillian Mead in European Stroke Journal
