# Supplementary material for: A systematic review of current national hospital-based stroke registries monitoring access to evidence-based care and patient outcomes
Source: Eur Stroke J. 2025 Jan 21;10(3):646–54. doi: 10.1177/23969873241311821 (PMC11752151; doi:10.1177/23969873241311821)
Supplement: sj-docx-1-eso-10.1177_23969873241311821 – Supplemental material for A systematic review of current national hospital-based stroke registries monitoring access to evidence-based care and patient outcomes [file sj-docx-1-eso-10.1177_23969873241311821.docx]

# **Supplementary Material**

Table 1a. Literature Search Strategy - Ovid Medline

Ovid MEDLINE(R) and Epub Ahead of Print, In-Process, In-Data-Review & Other Non-Indexed Citations, Daily and Versions <1946 to December 07, 2023> Searched on 29 January 2024

| 1 | Ischemic Attack, Transient/ | 22077 |
| --- | --- | --- |
| 2 | exp Cerebral Hemorrhage/ | 38377 |
| 3 | exp Stroke/ | 178385 |
| 4 | ((ischemic adj2 (attack or stroke)) or acute stroke).ti,ab,kf. | 89356 |
| 5 | 1 or 2 or 3 or 4 | 254823 |
| 6 | Registries/ | 110070 |
| 7 | (((national or central*) adj5 stroke adj5 regist*) or (stroke and audit)).ti,ab,kf. | 1440 |
| 8 | ((stroke and (Internet or web)) adj2 data collection).ti,ab,kf. | 85 |
| 9 | 5 and 6 | 5697 |
| 10 | 7 or 8 or 9 | 6922 |
| 11 | limit 10 to dt=20150521-20240101 | 3944 |

Table 1b. Literature Search Strategy - Ovid Embase

Embase Classic+Embase <1947 to 2023 December 12> Searched on 29 January 2024.

| # | Search string | hits | comments |
| --- | --- | --- | --- |
| 1 | Ischemic attack, Transient/ | 22830 |  |
| 2 | exp Cerebral Hemorrhage/ | 190516 |  |
| 3 | exp Stroke/ | 331316 |  |
| 4 | ((ischemic adj2 (attack or stroke)) or acute stroke).ti,ab,kf. | 152241 |  |
| 5 | 1 or 2 or 3 or 4 | 560942 |  |
| 6 | Registries/ | 80836 |  |
| 7 | (((national or central*) adj5 stroke adj5 regist*).mp or (stroke and audit).ti,ab,kf. | 3954 | Freetext string to capture records without register subject heading |
| 10 | ((stroke and (Internet or web)) adj2 data collection).ti,ab,kf. | 112 |  |
| 11 | 5 and 6 | 4860 |  |
| 12 | 7 or 8 or 9 | 8676 |  |
| 13 | Limit to 10 to dd=20150521-20240101 | 3652 |  |
| 15 | (Stroke in Bahrain: rising incidence, multiple risk factors, and suboptimal care).m_titl. | 1 | Lines 15-43 are the included papers from prior review, used here to test the search is working as needed |
| 16 | (Countrywide stroke incidence, subtypes, management and outcome in a multiethnic Asian population: the Singapore Stroke Registry).m_titl. | 1 |  |
| 17 | (In-hospital stroke: characteristics and outcomes).m_titl. | 1 |  |
| 18 | Implementing a simple care bundle is associated with improved outcomes in a national cohort of patients with ischemic stroke.m_titl. | 2 |  |
| 19 | Can a novel clinical risk score improve pneumonia prediction in acute stroke care? A UK multicenter cohort study.m_titl. | 1 |  |
| 20 | "The quality of acute stroke care- an analysis of evidence-based indicators in 260 000 patients".m_titl. | 0 |  |
| 21 | Thrombolysis in patients with mild stroke: results from the Austrian Stroke Unit Registry.m_titl. | 2 |  |
| 22 | Current status of acute stroke management in Korea: a report on a multicenter, comprehensive acute stroke registry.m_titl. | 1 |  |
| 23 | Quality of acute ischemic stroke care in Thailand: a prospective multicenter countrywide cohort study.m_titl. | 1 |  |
| 24 | "National Stroke Registry (NSR) Terengganu and Seberang Jaya experience".m_titl. | 1 |  |
| 25 | "Cerebral venous thrombosis in a Mexican multicenter registry of acute cerebrovascular disease the RENAMEVASC study.".m_titl. | 1 |  |
| 26 | "The Registry of Canadian Stroke Network an evolving methodology.".m_titl. | 2 |  |
| 27 | (The China National Stroke Registry for patients with acute cerebrovascular events design, rationale, and baseline patient characteristics).m_titl. | 1 |  |
| 28 | (The first Mexican multicenter register on ischaemic stroke the PREMIER study demographics, risk factors and outcome).m_titl. | 1 |  |
| 29 | "Get With the Guidelines-Stroke performance indicators surveillance of stroke care in the Taiwan Stroke Registry Get With the Guidelines-Stroke in Taiwan.".m_titl. | 1 |  |
| 30 | Stroke monitoring on a national level PERFECT Stroke, a comprehensive, registry-linkage stroke database in Finland.m_titl. | 1 |  |
| 31 | (Protocol and pilot data for establishing the Australian Stroke Clinical Registry).m_titl. | 1 |  |
| 32 | Stroke working toward a prioritized world agenda.m_titl. | 3 |  |
| 33 | "Quality of ischemic stroke care in emerging countries the Argentinian National Stroke Registry (ReNACer)".m_titl. | 1 |  |
| 34 | (Hospital treatment of patients with ischemic stroke or "transient ischemic attack using the Get With The Guidelines program").m_titl. | 1 |  |
| 35 | Comparison of stroke ward care versus mobile stroke teams in the Hungarian stroke database project.m_titl. | 1 |  |
| 36 | "International experience in stroke registries Korean Stroke Registry.".m_titl. | 1 |  |
| 37 | International experience in stroke registry Japanese Stroke Databank.m_titl. | 1 |  |
| 38 | The Paul Coverdell National Acute Stroke Registry initial results from four prototypes.m_titl. | 1 |  |
| 39 | (Acute ischemic stroke care and outcome in centers participating in the Polish National Stroke Prevention and Treatment Registry).m_titl. | 1 |  |
| 40 | Duration of hospital participation in a nationwide stroke registry is associated with improved quality of care.m_titl. | 1 |  |
| 41 | Riks-stroke - a Swedish national quality register for stroke care.m_titl. | 1 |  |
| 42 | (Registration of acute stroke validity in the Danish Stroke Registry and the Danish National Registry of Patients).m_titl. | 1 |  |
| 43 | 15 or 16 or 17 or 18 or 19 or 20 or 21 or 22 or 23 or 24 or 25 or 26 or 27 or 28 or 29 or 30 or 31 or 32 or 33 or 34 or 35 or 36 or 37 or 38 or 39 or 40 or 41 or 42 | 32 | Test papers, with some duplicates |
| 44 | 12 and 43 | 24 | How many of the test papers are retrieved in the search with line 7 |
| 45 | 43 not 44 | 8 (includes 2 duplicates) | Test papers missing with this version of the search (6 total) |
| 46 | 13 and 43 | 26 | Testing with line 8 |
| 47 | 43 not 46 | 6 (1 dup) | 5 missing |
| 48 | 14 and 43 | 30 | Testing with line 9 |
| 49 | 43 not 48 | 2 | 2 missing |

Table 1c. Literature Search Strategy - Ovid Global Health

Global Health <1973 to 2024 Week 03>Searched on 29 January 2024.

| 1 | Ischemic Attack, Transient.mp. | 1 |
| --- | --- | --- |
| 2 | Cerebral Hemorrhage.mp. | 565 |
| 3 | exp Stroke/ | 17503 |
| 4 | ((ischemic adj2 (attack or stroke)) or acute stroke).ti,ab,hw. | 6450 |
| 5 | 1 or 2 or 3 or 4 | 18955 |
| 6 | Registries.mp. | 7152 |
| 7 | (((national or central*) adj5 stroke adj5 regist*).mp. or (stroke and audit)).ti,ab,hw. | 126 |
| 8 | ((stroke and (Internet or web)) adj2 data collection).ti,ab,hw. | 11 |
| 9 | 5 and 6 | 165 |
| 10 | 7 or 8 or 9 | 290 |
| 11 | limit 10 to yr=2015-2024 | 188 |

Table 1d. Literature Search Strategy – WHO Global Index Medicus

World Health Organisation regional indices in Global Index Medicus. Searched on 29 January 2024.

| 1 | (ischemic attack, transient) | 1051 |
| --- | --- | --- |
| 2 | (cerebral hemorrhage) | 7009 |
| 3 | (stroke) | 28989 |
| 4 | (((ischemic adj2 (attack OR stroke)) OR acute stroke)) | 28989 |
| 5 | ((tw:(tw:(ischemic attack, transient) )) OR (tw:(tw:(((ischemic adj2 (attack OR stroke)) OR acute stroke)))) OR (tw:(tw:(cerebral hemorrhage) )) OR (tw:(tw:(stroke)))) | 34706 |
| 6 | (registries) | 2304 |
| 7 | (((national OR central*) adj5 stroke adj5 regist*) OR (stroke AND audit)) | 54 |
| 8 | (((stroke AND (internet OR web)) data collection)) | 6 |
| 9 | ((tw:(tw:(registries) )) AND (tw:(tw:((tw:(tw:(ischemic attack, transient) )) OR (tw:(tw:(((ischemic adj2 (attack OR stroke)) OR acute stroke)))) OR (tw:(tw:(cerebral hemorrhage) )) OR (tw:(tw:(stroke))))))) | 174 |
| 10 | ((tw:(tw:((tw:(tw:(registries) )) AND (tw:(tw:((tw:(tw:(ischemic attack, transient) )) OR (tw:(tw:(((ischemic adj2 (attack OR stroke)) OR acute stroke)))) OR (tw:(tw:(cerebral hemorrhage) )) OR (tw:(tw:(stroke))))))))) OR (tw:(tw:(((stroke AND (internet OR web)) data collection)) )) OR (tw:(tw:(((national OR central*) adj5 stroke adj5 regist*) OR (stroke AND audit)) ))) | 230 |
| 11 | limit 10 to yr=2015-2024 | 108 |

Table 2 Reasons for Registry Exclusion

Registries that were identified in the literature search but were excluded for the reasons outlined in the table.

| Reasons for Exclusion |
| --- |
| Not National |
| Catalonia (Spain), Nanjing (China), Cornell (USA), Florida/Puerto Rico, Upper Austria, Northern French Alps, Dijon (France), Tokyo (Japan), Rhone Area (France), Lausanne (Switzerland), Latin America, Chile, Fukokua (Japan), Akita (Japan), Västroke (Sweden), Ontario (Canada), Indonesia, Ghana, Saudi Arabia, Moldova, SUN4P (Greece), RES-Q (Greece), Mongolia, Sierra Leone, Guinea, Germany, Guadelopue, Nigeria, Stay alive acute stroke registry, Indo US stroke registry |
| Data Collection Ended Before 2016 |
| Iceland, China-QUEST, J-MUSIC (Japan), JSSRS (Japan) (these were not included in 2016)  Poland, Bahrain, Argentina, Thailand (these had been included in 2016) |
| Narrow Population of Participants |
| Estonia, OPTIC, PERFORM |
| Only for Endovascular Treatment/ Large Vessel Occlusion |
| MR CLEAN, STRATIS, INTERICS (China), New Zealand, MERR (Mexico), CSPPC (China), GSR (Germany), National Registry for Interventional Treatment in Ischemic Stroke (Romania) |
| Wrong Study Design |
| Vietnam, J-ASPECT (Japan), CASTOR (China) |
| Not Stroke Specific |
| Germany |
| No Evidence Found |
| Russia |

**Table 3**

**Registries included in 2016 review compared to 2024 review**

Column 1 lists registries only included in the 2016 review that have been excluded from this ‘2024’ review for various reasons. Column 2 contains registries from the 2016 review with evidence of ongoing data collection since 2016 that were consequently included in this 2024 review. Column 3 shows existing registries established before 2016 but only included in 2024 review and the reasons for this. Column 4 comprises registries established during or since 2016 thus are only included in the 2024 review.

The reason for exclusion in column 1 is the primary reason found and there may be more reasons than one. Additional references are cited here in the table; access to Covidence is available on request, which contains the full list of references.

| **2016 review registries excluded from 2024 review**  **(reasons for exclusion)** | **Registries included in both reviews** | **Previously established registries included in only the 2024 review (reasons)** | **Registries established during/ since 2016 and included in only the 2024 review** |
| --- | --- | --- | --- |
| Ethos (USA)  Data only from 1999 to 2003 [(Hills and Johnston, 2006)](https://www.zotero.org/google-docs/?8n3Zbu) | Australian Stroke Clinical Registry (AuSCR) | Australian National Stroke Audit  The audit includes rehabilitation data as well as acute stroke care data. Therefore, is not just rehabilitation data [(‘national-stroke-audit-methodology.pdf’, no date)](https://www.zotero.org/google-docs/?j19SiY) | Czech Republic’s RES-Q |
| Hungarian Stroke Database Project  Only active under this name from 1997 to 1998 [(Óváry *et al.*, 2007)](https://www.zotero.org/google-docs/?HwYI5q) | Austrian Stroke Unit Registry | Barbados National Stroke Registry  BNR-Stroke is a component of the wider Barbados National Registry for Chronic Non-communicable Disease containing other diseases like cancer however the stroke database itself only includes stroke patients [(Harewood-Marshall *et al.*, 2018)](https://www.zotero.org/google-docs/?iBTl7m) | Hungary's RES-Q |
| Japanese Standard Stroke Registry  Only active from 2000 to 2007 [(Matsuo, 2023)](https://www.zotero.org/google-docs/?CcaZxy) | Danish Stroke Registry | Dutch Acute Stroke Audit (DASA) (Netherlands)  First year of audit was in 2014 therefore data may not have been released before 2016 review was published [(Kuhrij *et al.*, 2018)](https://www.zotero.org/google-docs/?SIV2rL) | Romania’s  RES-Q |
| Polish Hospital Stroke Registry  No evidence of data past 2013 - data now taken from Polish National Health Fund [(Chwojnicki *et al.*, 2018; Jermakow *et al.*, 2022)](https://www.zotero.org/google-docs/?uJwDMy) | Finnish Stroke Database (PERFECT Stroke) | Israel’s National Stroke Registry (INSR)  Established in 2014 therefore data may not have been released before 2016 review was published [(*National Stroke Registry*, no date)](https://www.zotero.org/google-docs/?rMcL3d) |  |
| Registry of the Canadian Stroke Network11 (now the Ontario Stroke Registry)  Regional Registry (only covers province of Ontario) [(Kapral *et al.*, 2016)](https://www.zotero.org/google-docs/?wm0Hch) | German Stroke Register Study Group (ADSR) | Israel’s National Acute Stroke Survey (NASIS)  Survey conducted every 3 years for 2 months since 2004. May appear there is not a full year of data collection however the sum of data since 2004 spans past 1 year [(Pollak *et al.*, 2017)](https://www.zotero.org/google-docs/?AkcrOt) | China’s Stroke Center Alliance Programme (CSCA) |
| Argentinian National Stroke Registry (ReNACer)  No evidence of data since 2006; cross sectional data for two years; three publications (10.1161/STROKEAHA.108.521062;  10.1016/j.jstrokecerebrovasdis.  2011.02.018 10.1016/j.jstrokecerebrovasdis.2013.03.009), (personal communication Luciano Sposato) | Get With the Guidelines (USA) | Japan’s Stroke Databank (JSDB)  Data is referred to as highly representative at a national level therefore should be included in this review [(Matsuo, 2023)](https://www.zotero.org/google-docs/?RcFN5m) | India’s National Stroke Registry Programme funded by Indian Council of Medical Research |
| [Bahrain No evidence of data since 2011](https://www.zotero.org/google-docs/?FPxkR5) [[(Banna](https://www.zotero.org/google-docs/?FPxkR5) *[et al.](https://www.zotero.org/google-docs/?FPxkR5)*[, 2015)](https://www.zotero.org/google-docs/?FPxkR5)](https://www.zotero.org/google-docs/?Tt99i1) | Korean Stroke Registry | Norwegian Stroke Registry (NSR)  Referred to as a ‘nation wide’ quality registry so included  [(Søyland *et al.*, 2024)](https://www.zotero.org/google-docs/?YVZNYF)  The NSR is part of a wider cardiovascular disease registry, however the NSR itself only contains stroke data  [(*Overview of health registries at FHI*, 2024)](https://www.zotero.org/google-docs/?eYPhRj) | Kyrgyzstan’s RES-Q |
| PREMIER study (Mexico)  No evidence of data since 2006 [(Ruiz-Sandoval *et al.*, 2018)](https://www.zotero.org/google-docs/?stYCqX) | National Stroke Register/Audit of Ireland | Qatar’s Stroke Registry Of Hamad’s General Hospital  Stroke database was initiated in 2014 therefore data may not have been released before publishing of the 2015 review [(Imam *et al.*, 2020)](https://www.zotero.org/google-docs/?I6wq5R) | Nepal’s RES-Q |
| RENAMEVASC study (Mexico)  No evidence of data since 2004 [(Ruiz-Sandoval *et al.*, 2012)](https://www.zotero.org/google-docs/?u27xp3) | Paul Coverdell National Acute Stroke Registry (USA) | Slovakia’s Stroke register at the National Health Information Center | Sri Lanka’s Stroke Clinical Registry |
| Russian National Stroke Registry  No evidence of data since 2014 [(Stakhovskaya *et al.*, 2016)](https://www.zotero.org/google-docs/?eYj5Of) | Scottish Stroke Care Audit | Spain’s National Stroke Registry Of The Spanish Society Of Neurology (RENISEN)  Registry referred to as ‘national stroke registry’ therefore included | Uzbekistan’s National Stroke Registry |
| Thai Stroke Registry  No evidence of data since 2010 [(Nilanont *et al.*, 2014)](https://www.zotero.org/google-docs/?haGGrE) | Sentinel Stroke National Audit Programme (UK – England, Northern Ireland, Wales) | Switzerland’s Stroke Registry (SSR) |  |
|  | Singapore Stroke Registry | China’s Bigdata Observatory Platform for Stroke |  |
|  | Sweden’s national register |  |  |
|  | Taiwan Stroke Registry |  |  |
|  | China National Stroke Registry - Now known as Third China National Stroke Registry (CNSR-III) |  |  |
|  | National Stroke/ Neurology Registry (Malaysia) |  |  |
|  | Clinical Research Center for Stroke – fifth Division (CRC-5) registry (South Korea)  Previous name for Korean Stroke Registry |  |  |
| **Total = 11 Registries** | **Total = 17 Registries listed but actually there the two from Korea are part of the same registry, so we have listed 16 registries in the paper text** | **Total = 12 Registries** | **Total = 9 Registries** |

**Table 4**

**Funding, management, governance, hospital participation and coverage of registries**

Column 1: This name is used on an official registry website, an official annual report or appears in most publications.

Column 2: These dates refer to the start date of the registry and when the most recent report/ data has been released/ evidenced, NOT the registry end date. For RES-Q registries, the start date refers to the date the RES-Q data was made the national registry, if this is unspecified the date the country first added data to RES-Q is reported. Also for RES-Q country data the funding and managing organisation of the international RES-Q is reported.

‘Unspecified’ indicates where information couldn’t be retrieved.

| **Country** | **Funding** | **Managing Organisation** | **Governance** | **Voluntary/mandated participation and number of hospitals** | **Population Coverage** |
| --- | --- | --- | --- | --- | --- |
| **Australia: Australian National Stroke Audit** | **Community and industry** | **Non-profit organisation**: The Stroke Foundation | **Clinical council**: providing advice and governance  [(‘national-stroke-audit-methodology.pdf’, no date)](https://www.zotero.org/google-docs/?i14rzZ) | **Voluntary**  **107 hospitals** | **89% of acute stroke admissions** |
| **Australian stroke clinical registry (AUSCR)** | **Government (primarily), industry and non profit**  Annual funding income = **$1,054,147** (as of 2022) | **Consortium** of academic research institutes, an academic society and non profit organisations | **Steering committee** (for governance)  **Management committees** (for everyday operations) | **Voluntary**  [(‘Hospital Participation’ 2017)](https://www.zotero.org/google-docs/?8VJBND)  **61 hospitals (in** 2022), covering 75% acute stroke hospitals | Unspecified |
| **Austria** | **Government** | **Government** (Federal Ministry of Health) | Unspecified | **Mandatory participation** of hospitals with a stroke unit  **38 stroke units** (as of 2019) | **Covers ⅔ of all acute strokes** admitted to hospital |
| **Barbados** | **Government** | **Government initiative**  Research conducted by academic research institute | **Professional advisory board** | **Single public tertiary care hospita**l (Queen Elizabeth Hospital) managing a large proportion of Barbados’ stroke individuals, **and community sources** providing data | Unspecified |
| **Czech Republic’s RES-Q** | **Non-profit scientific society** (ESO)  And unrestricted grants from industry  (BI Angels Initiative, IPSEN, AstraZeneca) | **Academic institute**:  developed and maintained by Czech Republic’s Health Management Institute (HMI) with guidance from ESO (non-profit organisation)  Became a national registry under leadership of Czech stroke society | **Scientific committee**  [(*Scientific committee*, no date)](https://www.zotero.org/google-docs/?0BBIOZ) | **Voluntary participation**  **52 hospitals** (as of 2022)  [(*Hospitals using RES-Q*, no date)](https://www.zotero.org/google-docs/?c8hBp8) | Unspecified |
| **Denmark** | **Political organisation**Danish regions | **Public organisation**: The Danish Clinical Quality Program– National Clinical Registries (RKKP)  Regulated by Government | **Steering group** | **Mandatory participation** for all hospitals departments treating stroke | Unspecified |
| **Finland** | **Government**  Finnish institute for health and welfare | **Government**  Finnish institute for health and welfare | Managing group | **Mandatory participation**  24 hospitals/hospital groups (as of 2021) | 85% |
| **Netherlands** | **Industry:** From ‘Zorgverzekeraars Nederland’ (umbrella organisation of nine health insurers in the Netherlands) | **Scientific society** (Netherlands society of Neurology)  Facilitated by non-profit organisation: Dutch Institute for Clinical Auditing (DICA) | **Clinical audit board** (mandated clinical experts in acute stroke care) | **Voluntary participation**  [(*Home*, no date)](https://www.zotero.org/google-docs/?4H2wwW)  **81 hospitals** (as of 2016) | Unspecified |
| **Germany** | Unspecified | **Non- profit association**: Working Group of German-Speaking Stroke Registers | **Multidisciplinary working group** including German Stroke Society, The German Society for Neurology and The German Stroke Aid Foundation representatives | **Voluntary participation** of **10 regional registries**  (participation of hospitals in regional registries is mandatory) | Unspecified |
| **Hungary RES-Q** | **Non-profit scientific society**: ESO  And unrestricted grants from **industry**: BI Angels Initiative, IPSEN, AstraZeneca | **Academic institute**: developed and maintained by Czech Republic’s Health Management Institute (HMI)  Guidance from non-profit organization (ESO) | **Scientific committee**  [*Scientific committee*](https://www.zotero.org/google-docs/?XKUNO0) | **Voluntary participation**  **33 hospitals** (as of 2023) | Unspecified |
| **Ireland** | **Government health service**: Health Service Executive (HSE) | **Health service**: Under The National Office of Clinical Audit (NOCA) | **Steering group**  [(Kennedy *et al.*, 2022)](https://www.zotero.org/google-docs/?48FfDd) | **Voluntary Participation**  **22 hospitals** (as of 2022) | **90.1% coverage** of cases submitted by participating hospitals (as of 2022) - down from 95% (in 2021) |
| **Israel (NASIS)** | **Government** (Israeli Center for Disease Control under Israeli Ministry of Health)  And **Industry** eg. Novo-Nordisk, Pfizer, Sanofi-Aventis, Rafa Laboratories, and Teva | **Government** and **scientific association** (Israel Neurological Association) | Unspecified | **Mandatory participation**  Covers all **28 hospitals** in Israel | Unspecified |
| **Israel (INSR)** | Unspecified | **Government** (Israel Center for Disease Control at the Ministry of Health) | Unspecified | **27 general hospitals** (as of 2020) | Unspecified |
| **Japan** | **Government** Japan Agency for Medical Research and Development  Aimed to receive funding from academic societies too | **Government**  Facilitated by **government hospital/ research institute** | **Steering committee** of stroke medical researchers in Japan | **132 hospitals** (as of 2021) | Unspecified |
| **South Korea** | **Government**National institute of health/ Korea Disease Control and Prevention Agency/ South Korean Ministry of Welfare and Health | **Scientific society:** Korean Stroke Society | **Steering committee** (established in 2016) | **89 hospitals** (as of 2023) | Unspecified |
| **Norway** | **Government health service**  Norwegian institute of public health | **Government**  Norwegian institute of public health | **Professional advisory group** | **100% Adherence from all hospitals** | **87% coverage** (as of 2018) |
| **Qatar** | Unspecified | **Health service:** Hamad medical corporation (hospital) | Unspecified | **1 hospital**: Hamad general hospital, the sole provider of acute stroke care in Qatar  Hamad general hospital covers 90% of all stroke admissions | Unspecified |
| **Romania**  **RES-Q** | **Non-profit scientific society**: ESO  And unrestricted grants from **industry**: BI Angels Initiative, IPSEN, AstraZeneca | **Academic institute**: developed and maintained by Czech Republic’s Health Management Institute  Guidance from non-profit organization (ESO) | **Scientific committee** | **Voluntary participation**  **43 hospitals** (as of 2022) | Unspecified |
| **Scotland** | **Government**  Received annually from Public Health Scotland | **Government health service** Public Health Scotland | **Steering group** for strategic direction and clinical input | **Mandatory participation**  All hospitals providing stoke care | Unspecified |
| **Singapore** | **Government**  The Ministry of Health | **Government**: National registry of disease offices under The Ministry of Health | **Multi-**  **disciplinary Honorary Advisory Committee** provides advice - comprised of university, public and private stroke physicians | **All public hospitals** (as of 2023) | **94% of national stroke episodes** |
| **Slovakia** | **Government** National Health Information Centre (funded by Slovakia’s Ministry Of Health) | **Government**: (National Health Information Centre) | Unspecified | **Mandatory participation** of all primary and secondary stroke centers  43 hospitals (as of 2020) | Unspecified |
| **Spain** | **Scientific Society:** Stroke project of the Spanish Cerebrovascul-ar Diseases Study Group | **Scientific society**: The Spanish Society of Neurology | Unspecified | **35 hospitals** | Unspecified |
| **Sweden** | **Government** National Board of Health and Welfare and Sweden’s Municipalities and Regions | **Government Health Service**: Registercentru-m Norr, Region Västerbotten | **Steering group** | Initially voluntary but now **covers all hospitals** admitting patients with acute stroke  **72 hospitals** (as of 2021) | **95% coverage of all patients** treated with stroke in hospital (as of 2021) |
| **Switzerland** | **Non-profit organisations**: Switzerland Stroke Society and Swiss Heart Foundation | **Academic society:** Swiss Stroke Society | **Steering committee** of multidisciplinary researchers | **Mandatory participation** - All hospitalised patients in stroke units and comprehensiv-e stroke centres  **23 stroke units** (as of 2024) | Unspecified |
| **Taiwan’s Stroke Registry** | **Government**Ministry of Health and Welfare | **Academic society:** Taiwan stroke society | Unspecified | **Voluntary participation**  64 academic and community hospitals in Taiwan (as of 2020) | Unspecified |
| **United Kingdom (England, Wales & Northern Ireland)** | **Government health service:** funded by NHS England and the Welsh Government | **Independent organisation**: Healthcare Quality Improvement Partnership (HQIP)  **And a Consortium**: King’s College London and NHS England | **Steering group** | **Mandatory participation**All hospitals admitting acute stroke  **142 hospitals** in England, Wales and Northern Ireland (as of 2018) | **Covers ~95%** acute stroke hospital admissions |
| **USA (‘Get with the Guidelines’)** | Combination of **industry and non profit**: Eg. American Heart Association (AHA), American Stroke Association, Janssen Pharmaceutical Companies Johnson & Johnson | **Non-profit scientific society:** American Heart Association (AHA) | **Steering and Quality Improvement Committees** | **Voluntary participation**  **Over 2,000 hospitals** | Unspecified |
| **USA (Paul Coverdell National Acute Stroke Program)** | **Government**  Centers for Disease Control and Prevention (CDC) competitively funds the states  3 year recurring grant plan, last evidenced in 2020 | **Government**  Centers for Disease Control and Prevention | **Steering committee** | **Voluntary participation**  **13 participating states** (as of 2020)  **Almost 800 hospitals** (as of 2023) | Unspecified |
| **China’s Bigdata Observatory Platform for Stroke** | **Chinese government**  **838.4 million CNY** (Chinese Yuan) | **Government:** Ministry Of Health | **China Stroke Prevention Project Committee** (CSPPC) | **>1500 participating stroke centres** (as of 2020)    **31 out of 34 provinces** (as of 2020) of mainland China | Unspecified |
| China’s Stroke Center Alliance Programme (CSCA) | Unspecified | **Academic, non-profit organisation**: Chinese Stroke Association (CSA)  **Government guidance**: The National Center of Neurological Diseases Care Management | **Steering Committee** | **Voluntary participation**  Programme available to all secondary and tertiary hospitals  **1576 contributing hospitals** (as of 2017) | Unspecified |
| **China’s Third National Stroke Registry (CNSR-III)** | **Government** Ministry of Health and Ministry of Science and Technology | **Government**: Ministry of Health and Ministry of Science and Technology | **Steering Committee** | Hospitals selected by steering committee from **voluntary sites**  **201 hospitals** in 22 provinces and four municipalities (as of 2018) | Unspecified |
| **Malaysia** | **Government**Ministry of Health  Research grant | **Government,** ministry of health, with the National Clinical Research Centre (CRC) | **Steering committee:** subject matter experts from Ministry of Health and universities | **Voluntary participation** of state hospitals  **15 public hospitals** (as of 2020)  Covering **11 out of 13 Malaysian states** (as of 2019) | Unspecified |
| India | Indian registry is funded by Indian Council of Medical Research | **Government:** Indian Council of Medical Research-National Centre for Disease Informatics and Research | **Research area panel** on stroke and scientific advisory committee | **Voluntary participation**  **54 hospital based stroke registries** comprising 5 population based stroke registries (as of 2019) | Unspecified |
| Kyrgyzstan RES-Q | **Non-profit scientific society**: ESO  And unrestricted grants from **industry**: BI Angels Initiative, IPSEN, AstraZeneca | **Academic institute**: developed and maintained by Czech Republic’s Health Management Institute (HMI)  Guidance from non-profit organisation  (ESO) | **Scientific committee**  [(*Scientific committee*, no date)](https://www.zotero.org/google-docs/?uoY3zx) | **Voluntary participation**  **18 hospitals** (as of 2021) | Unspecified |
| **Nepal’s RES-Q** | **Non-profit scientific society**:  ESO and unrestricted grants from **industry**: BI Angels Initiative, IPSEN, AstraZeneca | **Academic institute**: developed and maintained by Czech Republic’s Health Management Institute (HMI)  Guidance from non-profit organization (ESO) | **Scientific committee** | **Voluntary participation**  **27 hospitals** (as of 2023) | Unspecified |
| Uzbekistan | Unspecified | Unspecified | Unspecified | Covers **12 regions** of the Republic of Uzbekistan | Unspecified |
| Sri Lanka | **Government grants**: (ministry of health Sri Lanka)  Other grants: **scientific association** (World Federation of Neurology and **non profit organisation** the World Stroke Organization (WSO) | **Government approved**: (ministry of health)  Operated by university and hospital of colombo | **Management committee** | Initial phase of the registry: **6 tertiary care hospitals out of 9 stroke units** in Sri Lanka | Unspecified |

**Table 5 supplementary materials (long version of table 1 in the main manuscript)**

| **Registry identified** | **Active dates** | **Data collection method** | **Consent** | **Follow-up** | **Targets/guidelines used to benchmark performance** | **Feedback to hospitals** | **Data Completeness** | D**ata privacy** | **Data linkage** |
| --- | --- | --- | --- | --- | --- | --- | --- | --- | --- |
| Australia: Stroke Clinical Registry) [7] | 2009- | Web-based, linked administrative data | Opt-out consent  Waiver of consent for in-hospital deaths | 90-180 days  Two mail attempts and phone call | National Best practice guidelines | Tailored reports and interactive data dashboards | Works with hospital staff to ensure data completeness | Anonymised | Yes |
| Australia: The Stroke Foundation National Audit of Acute Services [8] | 2007- | Web-based, linked administrative data | Consent forms for hospitals | NS | Australian national guidelines; *Acute Stroke Services Framework 2015* | Site-specific report highlighting performance | Manual reliability checks involving re-auditing 3–5 cases by a different auditor | Anonymised | Yes |
| Austria [9] | 2003- | Web-based data | Obligatory Immediate data collection | 3 month phone call | NS | NS | Recurrent online plausibility checks | Anonymised | NS |
| Barbados [10] | 2008- | Web-based data  Linked administrative data | NS | NS | Get With The Guidelines | NS | NS | Anonymised | Yes |
| China: Stroke Centre Alliance Programme [11] | 2015-2019 | Web-based data | No requirement for consent | No follow up | Programme steering Committee specifies national standards/guideline recommendations | Multiple on-demand reports showcasing performance measures using web-based tool | Certain variables are mandatory at data entry | Anonymised | No – has the potential |
| China: Bigdata Observatory Platform for Stroke [12] | 2011- | NS | NS | NS | NS | NS | NS | NS | NS |
| China: third National Stroke Registry [13] | 2015-2018 | Web-based data | Consent waivers | 3, 6 and 12 months; up to 5 years through electronic data capture system | NS | Feedback to researchers uploading data | Automated checks conducted to ensure the completeness of all data elements | Password protected electronic system | NS |
| Czech Republic (RES-Q) [14] | 2016- | Web-based data | NS | NS | ESO | Real time feedback on stroke care quality ( dashboards/reports, benchmarked against national average | NS | NS | Yes |
| Denmark [15] | 2003- | Web-based data  Linked administrative data | No requirement for consent | ‘Long’ follow up. | NS | NS | Yearly structured audit | NS | Yes |
| Germany [16] | 1999- | Web-based data  Linked administrative data | Implied that consent is not required Informed consent required for follow-up | NS | NS | NS | Quality assurance projects | NS | NS |
| Hungary (RES-Q) [17] | 2016- | Web-based data | NS | NS | ESO | Real time feedback on stroke care quality (dashboards and reports, providing benchmarking against national average | NS | NS | Yes |
| India [18] | 2018-2019 | Forms - Staff go to stroke centres/hospitals and collect data | NS | Up to 28 days  Revisiting hospitals, telephone interviews or house visits | NS | NS | Certain variables are mandatory at data entry.  Periodic audits | NS | NS |
| Ireland [19] | 2011- | Web-based data  Linked administrative data | NS | No follow up | Irish Heart Foundation, United Kingdom Royal College of Physicians 2016 guidelines | Feedback to hospitals; performance is measured against national standards | Data analyst assesses completeness  Reports include missing data | Anonymised | NS |
| Israel: National Acute Stroke Israeli Survey Registry [20] | 2004-2016 | Web-based data | Individual informed consent waiver | NS | NS | NS | NS | Anonymised | NS |
| Israel: The Israeli National Stroke Registry [21] | 2014- | Web-based data forms  Linked administrative data | Consent not required | NS | NS | NS | NS | Anonymised | Yes |
| Japan [22] | 1999- | Web-based data forms | Opt-out consent | NS | Own guidelines based on ESO and GWTG | Does not give feedback but aims to in the future | NS | Anonymised | No – aims to in the future |
| Kyrgyzstan (RES-Q) [23] | 2019- | Web-based RES-Q data forms | NS | NS | ESO guidelines | Real time feedback (dashboards and reports) for benchmarking against national average | NS | NS | Yes |
| Malaysia [24] | 2009- | Web-based data forms | NS | 3, 6 months standardised electronic case form | 2012 National Clinical guidelines | NS | NS | Anonymised | NS |
| Nepal (RES-Q)[25] | 2021- | Web-based RES-Q data | NS | NS | ESO | Real time feedback (dashboards and reports, for benchmarking against national average | NS | NS | Yes |
| Finland (PERFECT Stroke) [26] | 1999- | Linked administrative data | Informed consent not needed | Continuous (10+ years) | No target setting | Public benchmarking dashboards and reports with 1-2 year reporting lag | Validated hospital discharge data | Reports on aggregate level only, primary data pseudonymised by national authority | Yes |
| Netherlands [27] | 2014- | Web-based data  Linked administrative data | Waiver for patient consent | 3 months | Dutch evidence based guidelines | Results provided weekly to participating hospitals through a secure website | NS | Anonymised | Yes - some hospitals have data linkage |
| Norway [28] | 2007- | Web-based data  Paper forms | No consent required | 3 months | Norwegian National guideline | NS | Data quality checks | Anonymised data | NS |
| Qatar [29] | 2014- | NS | Informed consent is not required | 90 day follow up | ACC/AHA guideline | NS | Verified by patient records | NS | NS |
| Romania (RES-Q) [30] | 2017- | Web-based data | NS | NS | ESO | Real time feedback compared against national average | NS | NS | Yes |
| Scotland [31] | 2002- | Web-based data  Linked administrative data | Informed consent is not required; services expected to ensure participants are aware of data collection or to allow individuals to opt out | NS | Scottish Stroke Care Standards | Provides regular data to all NHS Boards | Data is cross-referenced with SMR01 data to confirm the absence of any overlooked patients | Anonymised | Yes |
| Singapore [32] | 2002- | Web-based data  Linked administrative data | Informed consent is not required | NS | NS | NS | NS | NS | NS |
| Slovakia [33] | 2010- | NS | Informed consent is not required | NS | ESO Quality parameters | NS | NS | Anonymised | NS |
| South Korea [34] | 2006- | Linked administrative data | Waiver of informed consent | 1, 3 and 12 months outpatient clinic visits or phone calls | NS | Mortality rate fed back to hospitals | NS | Anonymised | NS |
| Spain [35] | 2011-2019 | NS | NS | NS | NS | NS | NS | NS | NS |
| Sri Lanka  [36] | 2016-2017 | Web-based data forms using handheld device | Opt-out procedure informed consent done by providing information sheets and brochures | 3 month  telephone or/and email questionnaire | Own registry guidelines | Individual pre-specified reports of registry data given to each hospital | Data was checked for outliers and impossible data by trained staff | Password protected electronic system | NS |
| Sweden [37] | 1994- | Web-based data  Paper Forms | Opt-out procedure | 3, 12 month patient questionnaire | Swedish national guidelines and national targets | Realtime data dashboards for participating hospitals | Quality control measures are in place to verify the accuracy of personal identity numbers, gender, dates, etc | Password protected electronic system | Yes |
| Switzerland [38] | 2014- | Web-based data  Linked administrative data | NS | 3 months compulsory assessment | ESO | NS | NS | Anonymised | Yes |
| Taiwan [39] | 2006- | Web-based data | Signed informed consent forms | 1, 3, 6 months patient evaluation | NS | NS | Quality assurance projects | Anonymised | NS |
| UK - England, Wales & Northern Ireland [40] | 2013- | Web-based data  Linked administrative data | NS | 6 months  stroke review and follow up assessment | UK national guidelines | Provides minute-by-minute feedback on key indicators of care quality | NS | Anonymised | Yes |
| USA Get With The Guidelines [41] | 2003- | Web-based data | The AHA does not mandate informed consent, but local site human research committees may request it | 30 day follow-up form is optional for hospitals to complete. Patients do not consent to self report their outcomes. If the follow-up data are in the medical record, these are recorded in follow-up forms | AHA and American Stroke Association | GWTG-S provides realtime feedback, recognition and peer benchmarking which is a hallmark of the program | Structured data fields with logic checks ensure valid data are entered and recorded | NS | No |
| USA. Paul Coverdell National Acute Stroke Program [42] | 2001- | Web-based data forms | NS | 30 day follow up; method chosen by hospital | AHA and American Stroke Association | Feedback to states on adherence to care guidelines | Comprehensive mandatory columns in forms | NS | Yes |
| Uzbekistan [43] | 2019-2021 | NS | NS | NS | NS | NS | NS | NS | NS |

**Table 6. Patient characteristics collected listed by country and registry.**

Green = Upper income countries

Yellow = Upper middle Income Countries

Orange = Low middle income countries

Note that are no low income countries with registries

| National stroke register | Austria | Australia (AuSCR) | Australia (National Audit) | Barbados | China (CNSR-III) | China (CSCA) | China (BOSC) | Czech Republic | Denmark | Finland | Germany | Hungary | India | Ireland | Israel (NASIS) | Israel (INSR) | Japan | Kyrgyzstan | Malaysia | Nepal | Netherlands | Norway | Qatar |
| --- | --- | --- | --- | --- | --- | --- | --- | --- | --- | --- | --- | --- | --- | --- | --- | --- | --- | --- | --- | --- | --- | --- | --- |
| Age | ✔️ | ✔️ | ✔️ | ✔️ | ✔️ | ✔️ | ✔️ | ✔️ | ✔️ | ✔️ | ✔️ | ✔️ | ✔️ | ✔️ | ✔️ | ✔️ | ✔️ | ✔️ | ✔️ | ✔️ | ✔️ | ✔️ | ✔️ |
| Sex | ✔️ | ✔️ | ✔️ | ✔️ | ✔️ | ✔️ | ✔️ | ✔️ | ✔️ | ✔️ | ✔️ | ✔️ | ✔️ | ✔️ | ✔️ | ✔️ | ✔️ | ✔️ | ✔️ | ✔️ | ✔️ | ✔️ | ✔️ |
| Ethnicity |  | ✔️ | ✔️ |  | ✔️ | ✔️ | ✔️ |  |  |  |  |  |  |  | ✔️ | ✔️ |  |  | ✔️ |  |  |  | ✔️ |
| Stroke severity | ✔️ | ✔️ | ✔️ |  | ✔️ | ✔️ | ✔️ | ✔️ | ✔️ |  | ✔️ | ✔️ |  |  | ✔️ | ✔️ | ✔️ | ✔️ | ✔️ | ✔️ | ✔️ | ✔️ | ✔️ |
| Primary functional status | ✔️ | ✔️ | ✔️ | ✔️ | ✔️ |  | ✔️ | ✔️ |  |  | ✔️ | ✔️ |  | ✔️ | ✔️ |  | ✔️ | ✔️ |  | ✔️ |  | ✔️ |  |
| Previous stroke | ✔️ | ✔️ |  | ✔️ |  | ✔️ | ✔️ | ✔️ | ✔️ |  |  | ✔️ |  |  | ✔️ |  |  | ✔️ |  | ✔️ |  | ✔️ | ✔️ |
| Co-morbidity | ✔️ | ✔️ |  | ✔️ | ✔️ | ✔️ | ✔️ | ✔️ | ✔️ | ✔️ | ✔️ | ✔️ | ✔️ |  | ✔️ | ✔️ | ✔️ | ✔️ | ✔️ | ✔️ |  | ✔️ | ✔️ |
| Smoking | ✔️ |  |  | ✔️ | ✔️ | ✔️ | ✔️ | ✔️ | ✔️ |  | ✔️ | ✔️ | ✔️ |  | ✔️ |  |  | ✔️ | ✔️ | ✔️ |  | ✔️ | ✔️ |

| National stroke register | Romania | Scotland | Singapore | Slovakia | South Korea | Spain | Sri Lanka | Sweden | Switzerland | Taiwan | UK (not Scotland) | USA (GWTG) | USA (Paul Coverdell) | Uzbekistan |
| --- | --- | --- | --- | --- | --- | --- | --- | --- | --- | --- | --- | --- | --- | --- |
| Age | ✔️ | ✔️ | ✔️ |  | ✔️ | ✔️ | ✔️ | ✔️ | ✔️ | ✔️ | ✔️ | ✔️ | ✔️ | ✔️ |
| Sex | ✔️ | ✔️ | ✔️ |  | ✔️ | ✔️ | ✔️ | ✔️ | ✔️ | ✔️ | ✔️ | ✔️ | ✔️ | ✔️ |
| Ethnicity |  | ✔️ | ✔️ |  |  |  |  |  |  |  | ✔️ | ✔️ | ✔️ |  |
| Stroke severity | ✔️ |  |  | ✔️ | ✔️ | ✔️ |  | ✔️ | ✔️ | ✔️ |  | ✔️ | ✔️ | ✔️ |
| Primary functional status | ✔️ | ✔️ |  |  | ✔️ | ✔️ | ✔️ | ✔️ | ✔️ | ✔️ |  | ✔️ |  | ✔️ |
| Previous stroke | ✔️ |  |  |  | ✔️ | ✔️ | ✔️ |  | ✔️ | ✔️ | ✔️ | ✔️ | ✔️ |  |
| Co-morbidity | ✔️ |  | ✔️ |  | ✔️ | ✔️ | ✔️ | ✔️ | ✔️ | ✔️ | ✔️ | ✔️ | ✔️ | ✔️ |
| Smoking | ✔️ |  | ✔️ |  | ✔️ | ✔️ | ✔️ | ✔️ |  | ✔️ |  | ✔️ | ✔️ | ✔️ |

| National stroke register  **Table 7 Outcome measures by registry** | Austria | Australia (AuSCR) | Australia (National Audit) | Barbados | China (CNSR-III) | China (CSCA) | China (BOSC) | Czech Republic | Denmark | Finland | Germany | Hungary | India | Ireland | Israel (NASIS) | Israel (INSR) | Japan | Kyrgyzstan | Malaysia | Nepal | Netherlands | Norway | Qatar |
| --- | --- | --- | --- | --- | --- | --- | --- | --- | --- | --- | --- | --- | --- | --- | --- | --- | --- | --- | --- | --- | --- | --- | --- |
| Timing (months) | 3 | 6 |  | 1 | 3,6,  12,24 |  | 3,  12 | 3 | 3 | 1,3,12 | 3 | 3 | 1,3 | 0 | 3,12 | 1,  12 | 0 | 3 | 3 12 | 3 | 3 | 3 | 3 |
| Early neurological deterioration |  |  |  |  |  |  |  |  |  |  |  |  |  |  |  |  |  |  |  |  |  |  |  |
| Functional status | ✔️ | ✔️ |  |  | ✔️ |  | ✔️ | ✔️ | ✔️ |  | ✔️ | ✔️ |  | ✔️ | ✔️ |  | ✔️ | ✔️ | ✔️ | ✔️ | ✔️ | ✔️ | ✔️ |
| Living alone status |  | ✔️ |  |  |  |  |  |  | ✔️ |  |  |  |  |  |  |  |  |  |  |  |  |  |  |
| Management of comorbidities |  |  |  |  | ✔️ |  |  |  |  |  |  |  |  |  |  | ✔️ |  |  |  |  |  |  |  |
| Myocardial infarction |  |  |  |  |  |  |  |  |  |  |  |  |  |  |  |  |  |  |  |  |  |  |  |
| Survivor status | ✔️ | ✔️ |  | ✔️ | ✔️ |  | ✔️ |  | ✔️ | ✔️ | ✔️ |  | ✔️ | ✔️ | ✔️ | ✔️ | ✔️ |  | ✔️ |  | ✔️ | ✔️ | ✔️ |
| Place of residence |  | ✔️ |  |  |  | ✔️ |  | ✔️ | ✔️ | ✔️ |  | ✔️ |  | ✔️ | ✔️ |  | ✔️ | ✔️ | ✔️ | ✔️ |  | ✔️ |  |
| Quality of life |  | ✔️ |  |  |  |  |  |  |  |  |  |  |  |  |  |  |  |  |  |  |  | ✔️ |  |
| Recurrent stroke since discharge |  | ✔️ |  |  | ✔️ |  | ✔️ | ✔️ |  | ✔️ |  | ✔️ |  |  |  |  |  | ✔️ | ✔️ | ✔️ |  |  |  |
| Readmission to hospital |  | ✔️ |  |  |  |  |  |  | ✔️ |  |  |  |  |  |  |  |  |  | ✔️ |  |  | ✔️ |  |
| Secondary stroke prevention medication |  |  |  |  | ✔️ |  |  |  |  | ✔️ |  |  |  |  |  |  |  |  |  |  |  |  |  |
| Support for mood, behaviour, cognition |  |  |  |  |  |  |  |  |  |  |  |  |  |  |  |  |  |  |  |  |  |  |  |
| Vascular events | ✔️ |  |  |  | ✔️ |  |  |  |  |  |  |  |  |  |  |  |  |  | ✔️ |  |  |  |  |
| Mortality register link |  | ✔️ |  |  |  |  |  |  | ✔️ | ✔️ |  |  | ✔️ |  |  |  |  |  |  |  |  |  |  |

| National stroke register | | Romania | Scotland | Singapore | Slovakia | South Korea | | Spain | | Sri Lanka | | Sweden | | | Switzerland | | Taiwan | UK (not Scotland) | USA (GWTG) | | USA (Paul Coverdell) | | | Uzbekistan | |  |  |  |  |  |  |  |  |
| --- | --- | --- | --- | --- | --- | --- | --- | --- | --- | --- | --- | --- | --- | --- | --- | --- | --- | --- | --- | --- | --- | --- | --- | --- | --- | --- | --- | --- | --- | --- | --- | --- | --- |
| Timing (months) | | 3 | 3 | 0 |  | 3 | | 3 | | 3 | | | 3 | | 3 | | 1,3,6 | 6 | 3 | | 1 | | |  | |  |  |  |  |  |  |  |  |
| Early neurological deterioration | |  |  |  |  | ✔️ | |  | |  | | |  | |  | |  |  |  | |  | | |  | |  |  |  |  |  |  |  |  |
| Functional status | | ✔️ |  |  |  | ✔️ | | ✔️ | | ✔️ | | | ✔️ | | ✔️ | | ✔️ | ✔️ | ✔️ | | ✔️ | | | ✔️ | |  |  |  |  |  |  |  |  |
| Living alone status | |  |  |  |  |  | |  | |  | | | ✔️ | |  | |  |  |  | |  | | |  | |  |  |  |  |  |  |  |  |
| Management of comorbidities | |  |  |  |  |  | |  | |  | | |  | |  | |  |  |  | |  | | |  | |  |  |  |  |  |  |  |  |
| Myocardial infarction | |  |  |  |  | ✔️ | |  | |  | | |  | |  | |  |  |  | |  | | |  | |  |  |  |  |  |  |  |  |
| Survivor status | |  | ✔️ | ✔️ |  | ✔️ | | ✔️ | | ✔️ | | | ✔️ | | ✔️ | | ✔️ |  |  | | ✔️ | | | ✔️ | |  |  |  |  |  |  |  |  |
| Place of residence | | ✔️ |  |  |  |  | |  | |  | | | ✔️ | | ✔️ | | ✔️ |  | ✔️ | | ✔️ | | |  | |  |  |  |  |  |  |  |  |
| Quality of life | |  |  |  |  |  | |  | |  | | | ✔️ | |  | |  | ✔️ |  | |  | | |  | |  |  |  |  |  |  |  |  |
| Recurrent stroke since discharge | | ✔️ |  |  |  | ✔️ | |  | | ✔️ | | |  | | ✔️ | | ✔️ | ✔️ |  | | ✔️ | | |  | |  |  |  |  |  |  |  |  |
| Readmission to hospital | |  |  |  |  |  | |  | |  | | |  | |  | |  |  |  | | ✔️ | | |  | |  |  |  |  |  |  |  |  |
| Secondary stroke prevention medication | |  |  |  |  | ✔️ | |  | |  | | |  | |  | |  |  |  | |  | | |  | |  |  |  |  |  |  |  |  |
| Support for mood, behaviour, cognition | |  |  |  |  |  | |  | |  | | | ✔️ | |  | |  | ✔️ |  | |  | | |  | |  |  |  |  |  |  |  |  |
| Vascular events | |  |  |  |  | ✔️ | |  | |  | | |  | |  | |  |  |  | | ✔️ | | |  | |  |  |  |  |  |  |  |  |
| Mortality register link | |  | ✔️ | ✔️ |  |  | |  | |  | | |  | |  | |  |  |  | |  | | |  | |  |  |  |  |  |  |  |  |
|  | |  |  |  |  |  | |  | |  | | |  | |  | |  |  |  | |  | | |  | |  |  |  |  |  |  |  |  |
| Table 8. Investigations and treatments by registry  National stroke register | | | | | | Austria | | Australia (AuSCR) | | Australia (National Audit) | | | Barbados | | China (CNSR-III) | China (CSCA) | China (BOSC) | Czech Republic | Denmark | | Finland | Germany | | Hungary | | India | Ireland | Israel (NASIS) | Israel (INSR) | Japan | Kyrgyzstan | Malaysia | Nepal |
| **Ultra-acute:** | | | | | |  | |  | |  | | |  | |  |  |  |  |  | |  |  | |  | |  |  |  |  |  |  |  |  |
| Brain or vascular imaging | | | | | | ✔️ | | ✔️ | | ✔️ | | |  | | ✔️ |  |  | ✔️ | ✔️ | |  | ✔️ | | ✔️ | | ✔️ | ✔️ | ✔️ | ✔️ |  | ✔️ | ✔️ | ✔️ |
| Craniectomy | | | | | |  | |  | | ✔️ | | |  | | ✔️ | ✔️ | ✔️ | ✔️ |  | |  |  | | ✔️ | |  |  |  |  | ✔️ | ✔️ | ✔️ | ✔️ |
| Time of symptom onset | | | | | | ✔️ | | ✔️ | |  | | |  | |  | ✔️ | ✔️ | ✔️ |  | |  | ✔️ | | ✔️ | |  | ✔️ | ✔️ | ✔️ |  | ✔️ | ✔️ | ✔️ |
| Mode of admission | | | | | | ✔️ | | ✔️ | |  | | |  | | ✔️ | ✔️ | ✔️ | ✔️ | ✔️ | |  |  | | ✔️ | |  |  | ✔️ | ✔️ | ✔️ | ✔️ | ✔️ | ✔️ |
| Mechanical ventilation | | | | | |  | |  | |  | | |  | |  |  | ✔️ | ✔️ |  | |  |  | | ✔️ | |  |  |  |  |  | ✔️ | ✔️ | ✔️ |
| Onset to door Time | | | | | | ✔️ | | ✔️ | | ✔️ | | |  | |  | ✔️ | ✔️ | ✔️ | ✔️ | |  | ✔️ | | ✔️ | |  | ✔️ | ✔️ | ✔️ |  | ✔️ | ✔️ | ✔️ |
| Thrombolysis | | | | | | ✔️ | | ✔️ | | ✔️ | | | ✔️ | | ✔️ | ✔️ | ✔️ | ✔️ | ✔️ | | ✔️ | ✔️ | | ✔️ | |  | ✔️ | ✔️ | ✔️ |  | ✔️ | ✔️ | ✔️ |
| Door-to-needle time | | | | | | ✔️ | | ✔️ | | ✔️ | | |  | |  |  | ✔️ | ✔️ | ✔️ | |  | ✔️ | | ✔️ | |  | ✔️ |  | ✔️ |  | ✔️ | ✔️ | ✔️ |
| Time to neuroimaging | | | | | | ✔️ | | ✔️ | |  | | |  | |  |  |  | ✔️ | ✔️ | |  | ✔️ | | ✔️ | |  | ✔️ | ✔️ | ✔️ |  | ✔️ | ✔️ | ✔️ |
| Access to thrombectomy | | | | | |  | | ✔️ | | ✔️ | | |  | |  | ✔️ | ✔️ | ✔️ | ✔️ | |  | ✔️ | | ✔️ | |  | ✔️ |  | ✔️ |  | ✔️ | ✔️ | ✔️ |
| Door-to-groin puncture time | | | | | |  | | ✔️ | |  | | |  | |  | ✔️ |  | ✔️ | ✔️ | |  |  | | ✔️ | |  |  |  |  |  | ✔️ | ✔️ | ✔️ |
| Aggressive blood pressure lowering for intracerebral haemorrhage | | | | | |  | |  | |  | | |  | |  |  |  |  |  | |  |  | |  | |  |  |  |  |  |  | ✔️ |  |
|  | | | | | |  | |  | |  | | |  | |  |  |  |  |  | |  |  | |  | |  |  |  |  |  |  |  |  |
| **Ward care setting** | | | | | |  | |  | |  | | |  | |  |  |  |  |  | |  |  | |  | |  |  |  |  |  |  |  |  |
| Adequate fluid and nutrition | | | | | |  | |  | |  | | |  | |  |  |  |  |  | |  |  | |  | |  |  |  |  |  |  |  |  |
| Antithrombotic therapy during hospitalisation | | | | | |  | | ✔️ | |  | | | ✔️ | | ✔️ | ✔️ | ✔️ | ✔️ | ✔️ | |  | ✔️ | | ✔️ | |  | ✔️ |  | ✔️ | ✔️ | ✔️ | ✔️ | ✔️ |
| Early mobilisation | | | | | |  | | ✔️ | | ✔️ | | |  | |  |  |  |  | ✔️ | |  |  | |  | |  |  |  |  |  |  |  |  |
| Length of stay | | | | | | ✔️ | | ✔️ | |  | | |  | | ✔️ | ✔️ | ✔️ | ✔️ | ✔️ | | ✔️ | ✔️ | | ✔️ | | ✔️ | ✔️ | ✔️ | ✔️ |  | ✔️ | ✔️ | ✔️ |
| Rehabilitation | | | | | |  | | ✔️ | | ✔️ | | |  | | ✔️ |  | ✔️ | ✔️ | ✔️ | |  | ✔️ | | ✔️ | | ✔️ | ✔️ |  |  | ✔️ | ✔️ | ✔️ | ✔️ |
| Stroke unit care | | | | | | ✔️ | | ✔️ | | ✔️ | | |  | | ✔️ |  |  | ✔️ | ✔️ | |  | ✔️ | | ✔️ | |  | ✔️ | ✔️ |  |  | ✔️ | ✔️ | ✔️ |
| Stroke unit team management | | | | | | ✔️ | |  | | ✔️ | | |  | |  |  |  |  |  | |  |  | |  | |  |  |  |  |  |  |  |  |
| Treatment at stroke centre | | | | | |  | |  | | ✔️ | | |  | |  |  |  |  |  | | ✔️ |  | |  | |  | ✔️ |  |  |  |  |  |  |
|  | | | | | |  | |  | |  | | |  | |  |  |  |  |  | |  |  | |  | |  |  |  |  |  |  |  |  |
|  | | | | | |  | |  | |  | | |  | |  |  |  |  |  | |  |  | |  | |  |  |  |  |  |  |  |  |
| **Tests and Procedures:** | | | | | |  | |  | |  | | |  | |  |  |  |  |  | |  |  | |  | |  |  |  |  |  |  |  |  |
| Assessment for Rehabilitation | | | | | | ✔️ | | ✔️ | | ✔️ | | |  | |  | ✔️ |  |  | ✔️ | |  | ✔️ | |  | |  | ✔️ |  |  |  |  |  |  |
| Assessment of nutritional risk | | | | | |  | |  | | ✔️ | | |  | |  |  |  |  | ✔️ | |  |  | |  | |  | ✔️ |  |  |  |  |  |  |
| Carotid enterectomy/stenting | | | | | |  | |  | |  | | |  | | ✔️ |  |  | ✔️ | ✔️ | | ✔️ | ✔️ | | ✔️ | |  | ✔️ | ✔️ |  |  | ✔️ |  | ✔️ |
| Carotid imaging/ultrasound | | | | | |  | |  | |  | | |  | |  |  |  | ✔️ | ✔️ | |  | ✔️ | | ✔️ | |  | ✔️ | ✔️ | ✔️ |  | ✔️ | ✔️ | ✔️ |
| Dysphagia screening | | | | | |  | | ✔️ | |  | | |  | | ✔️ | ✔️ | ✔️ | ✔️ | ✔️ | |  | ✔️ | | ✔️ | |  | ✔️ |  |  | ✔️ | ✔️ | ✔️ | ✔️ |
|  | | | | | |  | |  | |  | | |  | |  |  |  |  |  | |  |  | |  | |  |  |  |  |  |  |  |  |
| **Secondary Prevention:** | | | | | |  | |  | |  | | |  | |  |  |  |  |  | |  |  | |  | |  |  |  |  |  |  |  |  |
| Discharged on an antihypertensive medication | | | | | |  | | ✔️ | | ✔️ | | |  | |  | ✔️ |  | ✔️ |  | | ✔️ |  | | ✔️ | |  |  | ✔️ |  | ✔️ | ✔️ | ✔️ | ✔️ |
| Discharged on an antithrombotic medication | | | | | |  | | ✔️ | | ✔️ | | | ✔️ | |  | ✔️ |  | ✔️ |  | | ✔️ | ✔️ | | ✔️ | |  | ✔️ | ✔️ |  | ✔️ | ✔️ | ✔️ | ✔️ |
| Discharge on statin/lipid-lowering medication | | | | | |  | | ✔️ | | ✔️ | | | ✔️ | |  | ✔️ |  | ✔️ |  | | ✔️ | ✔️ | | ✔️ | |  |  | ✔️ |  | ✔️ | ✔️ | ✔️ | ✔️ |
| Smoking cessation counselling | | | | | |  | |  | | ✔️ | | |  | |  | ✔️ |  | ✔️ |  | |  |  | | ✔️ | |  |  |  |  |  | ✔️ |  | ✔️ |
| **Long-term planning:** | | | | | |  | |  | |  | | |  | |  |  |  |  |  | |  |  | |  | |  |  |  |  |  |  |  |  |
| Access to rehabilitation after discharge | | | | | |  | | ✔️ | |  | | |  | |  |  |  |  | ✔️ | | ✔️ | ✔️ | |  | |  | ✔️ |  |  | ✔️ |  |  |  |
| Allied health assessment | | | | | |  | |  | |  | | |  | | ✔️ |  |  |  |  | |  |  | |  | |  | ✔️ |  |  |  |  | ✔️ |  |
| Continence plan | | | | | |  | |  | |  | | |  | |  |  |  |  |  | |  |  | |  | |  |  |  |  |  |  |  |  |
| Discharge care plan | | | | | |  | | ✔️ | | ✔️ | | |  | |  |  |  |  |  | |  |  | |  | |  | ✔️ |  |  |  |  |  |  |
| Educational (materials) provided | | | | | |  | | ✔️ | | ✔️ | | |  | |  | ✔️ |  |  |  | |  |  | |  | |  |  |  |  | ✔️ |  | ✔️ |  |
| Multidisciplinary meeting case asses | | | | | |  | |  | |  | | |  | |  |  |  |  |  | |  |  | |  | |  | ✔️ |  |  |  |  |  |  |
| Prognosis diagnosis discussed with relative/carer | | | | | |  | |  | |  | | |  | |  |  |  |  |  | |  |  | |  | |  |  |  |  |  |  |  |  |
| Referral to specialist neurovascular clinic | | | | | |  | |  | |  | | |  | |  |  |  |  |  | |  |  | |  | |  | ✔️ |  |  |  |  |  |  |

| National stroke register | Netherlands | Norway | Qatar | Romania | Scotland | Singapore | Slovakia | South Korea | Spain | Sri Lanka | Sweden | Switzerland | Taiwan | UK except Scotland | USA (GWTG) | USA (Paul Coverdell) | Uzbekistan |
| --- | --- | --- | --- | --- | --- | --- | --- | --- | --- | --- | --- | --- | --- | --- | --- | --- | --- |
| **Ultra-acute:** |  |  |  |  |  |  |  |  |  |  |  |  |  |  |  |  |  |
| Brain or vascular imaging |  |  | ✔️ | ✔️ | ✔️ |  | ✔️ | ✔️ | ✔️ | ✔️ | ✔️ | ✔️ | ✔️ | ✔️ | ✔️ | ✔️ | ✔️ |
| Craniectomy |  |  |  | ✔️ |  |  |  | ✔️ |  |  | ✔️ |  |  |  | ✔️ |  |  |
| Time of symptom onset |  |  |  | ✔️ | ✔️ |  | ✔️ | ✔️ |  |  | ✔️ | ✔️ |  | ✔️ | ✔️ | ✔️ |  |
| Mode of admission |  |  | ✔️ | ✔️ | ✔️ |  |  |  |  |  | ✔️ | ✔️ |  |  | ✔️ | ✔️ |  |
| Mechanical ventilation |  |  |  | ✔️ |  |  |  |  |  |  |  | ✔️ |  |  |  |  |  |
| Onset to door Time | ✔️ | ✔️ |  | ✔️ | ✔️ |  |  | ✔️ |  | ✔️ | ✔️ | ✔️ |  | ✔️ |  | ✔️ |  |
| Thrombolysis | ✔️ | ✔️ | ✔️ | ✔️ | ✔️ | ✔️ | ✔️ | ✔️ | ✔️ | ✔️ | ✔️ | ✔️ | ✔️ | ✔️ | ✔️ | ✔️ |  |
| Door-to-needle time | ✔️ | ✔️ | ✔️ | ✔️ | ✔️ | ✔️ | ✔️ | ✔️ |  | ✔️ | ✔️ | ✔️ |  | ✔️ | ✔️ | ✔️ |  |
| Time to neuroimaging |  |  |  | ✔️ | ✔️ |  |  |  |  | ✔️ |  | ✔️ |  | ✔️ | ✔️ | ✔️ |  |
| Access to thrombectomy | ✔️ | ✔️ | ✔️ | ✔️ | ✔️ |  | ✔️ | ✔️ | ✔️ |  | ✔️ | ✔️ |  | ✔️ | ✔️ |  |  |
| Door-to-groin puncture time |  |  |  | ✔️ | ✔️ |  |  | ✔️ |  |  |  | ✔️ |  |  | ✔️ |  |  |
| Aggressive blood pressure lowering |  |  |  |  |  |  |  |  |  |  |  |  |  | ✔️ | ✔️ |  |  |
|  |  |  |  |  |  |  |  |  |  |  |  |  |  |  |  |  |  |
| **Ward care setting** |  |  |  |  |  |  |  |  |  |  |  |  |  |  |  |  |  |
| Adequate fluid and nutrition |  |  |  |  |  |  |  |  |  |  |  |  |  |  |  | ✔️ |  |
| Antithrombotic therapy during hospitalisation |  |  |  | ✔️ | ✔️ | ✔️ |  | ✔️ |  |  | ✔️ | ✔️ |  | ✔️ | ✔️ | ✔️ |  |
| Early mobilisation |  |  |  |  |  |  |  |  |  |  |  |  |  |  |  |  |  |
| Length of stay |  | ✔️ | ✔️ | ✔️ |  |  |  | ✔️ | ✔️ | ✔️ | ✔️ | ✔️ |  |  | ✔️ | ✔️ |  |
| Rehabilitation |  |  |  | ✔️ |  |  |  |  |  | ✔️ | ✔️ |  |  | ✔️ | ✔️ |  |  |
| Stroke unit care | ✔️ | ✔️ | ✔️ | ✔️ | ✔️ |  |  |  | ✔️ | ✔️ | ✔️ | ✔️ |  | ✔️ | ✔️ |  |  |
| Stroke unit team management |  |  |  |  |  |  |  |  |  |  |  |  |  |  |  |  |  |
| Treatment at stroke centre |  |  |  |  | ✔️ |  |  |  |  |  | ✔️ | ✔️ |  | ✔️ |  |  |  |
|  |  |  |  |  |  |  |  |  |  |  |  |  |  |  |  |  |  |
| **Tests and Procedures:** |  |  |  |  |  |  |  |  |  |  |  |  |  |  |  |  |  |
| Assessment for Rehabilitation |  |  |  |  |  |  |  |  |  |  |  |  |  |  | ✔️ | ✔️ |  |
| Assessment of nutritional risk |  |  |  |  |  |  |  |  |  |  |  |  |  |  |  |  |  |
| Carotid enterectomy/stenting |  |  |  | ✔️ | ✔️ |  |  |  |  |  |  | ✔️ |  |  | ✔️ | ✔️ |  |
| Carotid imaging/ultrasound |  |  |  | ✔️ |  |  |  |  | ✔️ | ✔️ | ✔️ | ✔️ |  |  | ✔️ |  |  |
| Dysphagia screening |  | ✔️ |  | ✔️ | ✔️ |  |  |  |  | ✔️ | ✔️ |  |  | ✔️ | ✔️ | ✔️ |  |
|  |  |  |  |  |  |  |  |  |  |  |  |  |  |  |  |  |  |
| **Secondary Prevention:** |  |  |  |  |  |  |  |  |  |  |  |  |  |  |  |  |  |
| Discharged on an antihypertensive medication |  | ✔️ |  | ✔️ |  |  |  |  |  |  | ✔️ |  |  |  | ✔️ | ✔️ |  |
| Discharged on an antithrombotic medication |  | ✔️ |  | ✔️ |  |  |  | ✔️ |  | ✔️ | ✔️ |  | ✔️ |  | ✔️ | ✔️ |  |
| Discharge on statin/lipid-lowering medication |  | ✔️ |  | ✔️ |  |  |  |  |  | ✔️ | ✔️ |  | ✔️ |  | ✔️ | ✔️ |  |
| Smoking cessation counselling |  |  |  | ✔️ |  |  |  |  |  |  | ✔️ |  |  |  | ✔️ | ✔️ |  |
| **Long-term planning:** |  |  |  |  |  |  |  |  |  |  |  |  |  |  |  |  |  |
| Access to rehabilitation after discharge |  |  |  |  |  |  |  |  |  |  |  |  |  |  |  |  |  |
| Allied health assessment |  | ✔️ |  |  | ✔️ |  |  |  |  |  | ✔️ |  |  | ✔️ |  | ✔️ |  |
| Continence plan |  | ✔️ |  |  |  |  |  |  |  |  |  |  |  | ✔️ |  |  |  |
| Discharge care plan |  |  |  |  |  |  |  |  |  |  |  |  |  |  |  |  |  |
| Educational (materials) provided |  |  |  |  |  |  |  |  |  |  | ✔️ |  |  | ✔️ |  | ✔️ |  |
| Multidisciplinary meeting case asses |  |  |  |  |  |  |  |  |  |  | ✔️ |  |  |  | ✔️ | ✔️ | ✔️ |
| Prognosis diagnosis discussed with relative carer |  | ✔️ |  |  |  |  |  |  |  |  | ✔️ |  |  | ✔️ |  |  |  |
| Referral to specialist neurovascular clinic |  |  | ✔️ |  |  |  |  |  |  |  |  |  |  |  |  |  |  |
